# Diethyl 2,6-dimethyl-4-*p*-tolyl-1,4-dihydro­pyridine-3,5-dicarboxyl­ate

**DOI:** 10.1107/S1600536809035077

**Published:** 2009-09-05

**Authors:** Hoong-Kun Fun, Wei-Ching Liew, B. Palakshi Reddy, S. Sarveswari, V. Vijayakumar

**Affiliations:** a X-ray Crystallography Unit, School of Physics, Universiti Sains Malaysia, 11800 USM, Penang, Malaysia; b Organic Chemistry Division, School of Science and Humanities, VIT University, Vellore 632 014, India

## Abstract

In the title compound, C_20_H_25_NO_4_, the 1,4-dihydro­pyridine ring adopts a flattened-boat conformation and forms a dihedral angle of 89.77 (8)° with the benzene ring. Inter­molecular N—H⋯O hydrogen bonds result in the formation of extended chains parallel to the *b* axis.

## Related literature

For general background and applications of 1,4-dihydro­pyridine derivatives, see: Böcker & Guengerich (1986 ▶); Cooper *et al.* (1992 ▶); Vo *et al.* (1995 ▶); Gaudio *et al.* (1994 ▶); Gordeev *et al.* (1996 ▶); Sunkel *et al.* (1992 ▶). For ring conformations and ring puckering analysis, see: Boeyens (1978 ▶); Cremer & Pople (1975 ▶). For related 1,4-dihydro­pyridine structures, see: Fossheim *et al.* (1982 ▶); Teng *et al.* (2008 ▶); Bai *et al.* (2009 ▶). For the stability of the temperature controller used in the data collection, see: Cosier & Glazer (1986 ▶).
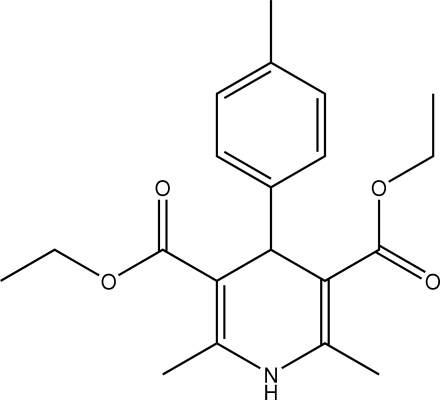


## Experimental

### 

#### Crystal data

C_20_H_25_NO_4_
*M* *_r_* = 343.41
Monoclinic, 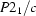

*a* = 10.0175 (1) Å
*b* = 7.4287 (1) Å
*c* = 25.0974 (3) Å
β = 105.528 (1)°
*V* = 1799.50 (4) Å^3^
*Z* = 4
Mo *K*α radiation
μ = 0.09 mm^−1^
*T* = 100 K
0.31 × 0.15 × 0.05 mm

#### Data collection

Bruker SMART APEXII CCD area-detector diffractometer
Absorption correction: multi-scan (**SADABS**; Bruker, 2005 ▶) *T*_min_ = 0.973, *T*_max_ = 0.996
20077 measured reflections
5309 independent reflections
3542 reflections with *I* > 2σ(*I*)
*R*_int_ = 0.046

#### Refinement

*R*[*F* ^2^ > 2σ(*F* ^2^)] = 0.057
*wR*(*F* ^2^) = 0.147
*S* = 1.02
5309 reflections
231 parameters
H-atom parameters constrained
Δρ_max_ = 0.33 e Å^−3^
Δρ_min_ = −0.26 e Å^−3^

### 

Data collection: *APEX2* (Bruker, 2005 ▶); cell refinement: *SAINT* (Bruker, 2005 ▶); data reduction: *SAINT*; program(s) used to solve structure: *SHELXTL* (Sheldrick, 2008 ▶); program(s) used to refine structure: *SHELXTL*; molecular graphics: *SHELXTL*; software used to prepare material for publication: *SHELXTL* and *PLATON* (Spek, 2009 ▶).

## Supplementary Material

Crystal structure: contains datablocks global, I. DOI: 10.1107/S1600536809035077/tk2533sup1.cif

Structure factors: contains datablocks I. DOI: 10.1107/S1600536809035077/tk2533Isup2.hkl

Additional supplementary materials:  crystallographic information; 3D view; checkCIF report

## Figures and Tables

**Table 1:** Hydrogen-bond geometry (Å, °)

*D*—H⋯*A*	*D*—H	H⋯*A*	*D*⋯*A*	*D*—H⋯*A*
N1—H1⋯O1^i^	0.84	2.15	2.9684 (18)	166

Symmetry code: (i) 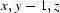.

**Fig. 1.**
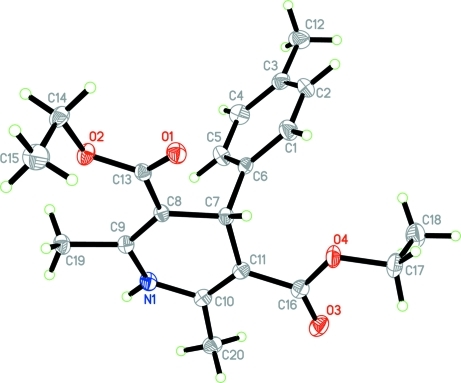
The molecular structure of (I), showing 50% probability displacement ellipsoids and the atom numbering scheme.

**Fig. 2.**
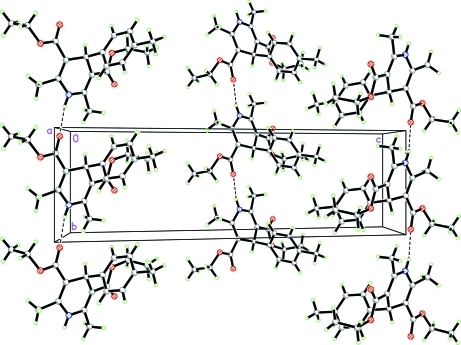
Partial unit cell contents for (I) highlighting the formation of supramolecular chains mediated by N-H···O hydrogen bonding (shown as dashed lines).

## supplementary crystallographic information

### Comment

Hantzsch 1,4-dihydropyridines (1,4-DHPS) are biologically active compounds which include various vasodilator, anti-hypertensive, bronchodilator, heptaprotective, anti-tumor, anti-mutagenic, geroprotective and anti-diabetic agents (Gaudio *et al.*, 1994). Nifedipine, Nitrendipine and Nimodipine *etc*., have found commercial utility as calcium channel blockers (Böcker & Guengerich, 1986; Gordeev *et al.*, 1996). For the treatment of congestive heart failure, a number of DHP calcium antagonists have been introduced (Sunkel *et al.*, 1992; Vo *et al.*, 1995). Some DHPs have been introduced as neuroprotectants and cognition enhancers. In addition, a number of DHPs with platelet anti-aggregatory activity have also been discovered (Cooper *et al.*, 1992).

In the title compound (I, Fig. 1), the bond lengths and angles have normal values and are comparable to closely related structures (Teng *et al.*, 2008; Bai *et al.*, 2009). The dihedral angle between the benzene ring and the mean plane of 1,4-dihydropyridine ring is 89.77 (8)°, indicating that both the rings are perpendicular to each other. The 1,4-dihydropyridine ring adopts a flattened-boat conformation (Boeyens, 1978; Cremer & Pople, 1975) with ring distortions at the nitrogen (Nl) and the tetrahedral carbon (C7). Both atoms are displaced in the same direction from the ring with distances of 0.115 (1) Å and 0.151 (2) Å, respectively, from the plane defined by C8, C9, C10, and C11, and form the apexes of a boat-type conformation (Fossheim *et al.*, 1982).

The crystal packing (Fig. 2) is consolidated by intermolecular N1—H1···O1 hydrogen bonds, Table 1, linking the molecules into chains parallel to the *b*-axis.

### Experimental

Compound (I) was prepared according to the Hantzsch pyridine synthesis. A mixture of *p*-tolylaldehyde (10 mmol), ethylacetoacetate (20 mmol) and ammonium acetate (10 mmol) were heated at 353 K for 2 h (monitored by TLC). After completion of the reaction, the mixture was cooled to room temperature and kept for 2 days to obtain the solid product. The solid that formed was washed using diethyl ether. Solid was collected separately and liquid was kept for solidification. The purity of the crude product was checked through TLC and recrystallized using acetone and ether. The compound was characterized by IR and ^1^H NMR. *M.p.* 383–385 K. IR (KBr):υ (cm^-1^), 3358, 2988, 1695, 1652, 1487, 1214;^1^H NMR (CDCl_3_): δ 1.23 (t, 6H, J = 8), δ 2.34 (s, 6H), δ 3.72 (s, 3H), δ 4.09 (m, 4H, J = 4), δ 4.94 (s, 1H), δ 5.58 (s, 1H), δ 6.76–7.21 (4H, aromatic).

### Refinement

The N-bound H atom was located in a difference Fourier map and constrained to ride with the parent atom with N-H = 0.84 Å, and with *U*_iso_(H)= 1.2 *U*_eq_(N). C-bound H atoms were positioned geometrically [C—H = 0.93–0.98 Å] and refined using a riding model, with *U*_iso_(H) = 1.2*U*_eq_(C) and 1.5*U*_eq_(methyl-C). A rotating group model was used for the methyl groups.

### Crystal data

**Table:**

C_20_H_25_NO_4_	*F*(000) = 736
*M**_r_* = 343.41	*D*_x_ = 1.268 Mg m^−^^3^
Monoclinic, *P*2_1_/*c*	Mo *K*α radiation, λ = 0.71073 Å
Hall symbol: -P 2ybc	Cell parameters from 3769 reflections
*a* = 10.0175 (1) Å	θ = 2.9–27.7°
*b* = 7.4287 (1) Å	µ = 0.09 mm^−^^1^
*c* = 25.0974 (3) Å	*T* = 100 K
β = 105.528 (1)°	Plate, colourless
*V* = 1799.50 (4) Å^3^	0.31 × 0.15 × 0.05 mm
*Z* = 4	

### Data collection

**Table:**

Bruker SMART APEXII CCD area-detector diffractometer	5309 independent reflections
Radiation source: fine-focus sealed tube	3542 reflections with *I* > 2σ(*I*)
graphite	*R*_int_ = 0.046
φ and ω scans	θ_max_ = 30.1°, θ_min_ = 2.1°
Absorption correction: multi-scan (*SADABS*; Bruker, 2005)	*h* = −14→14
*T*_min_ = 0.973, *T*_max_ = 0.996	*k* = −10→10
20077 measured reflections	*l* = −35→30

### Refinement

**Table:**

Refinement on *F*^2^	Primary atom site location: structure-invariant direct methods
Least-squares matrix: full	Secondary atom site location: difference Fourier map
*R*[*F*^2^ > 2σ(*F*^2^)] = 0.057	Hydrogen site location: inferred from neighbouring sites
*wR*(*F*^2^) = 0.147	H-atom parameters constrained
*S* = 1.02	*w* = 1/[σ^2^(*F*_o_^2^) + (0.0648*P*)^2^ + 0.5567*P*] where *P* = (*F*_o_^2^ + 2*F*_c_^2^)/3
5309 reflections	(Δ/σ)_max_ = 0.001
231 parameters	Δρ_max_ = 0.33 e Å^−^^3^
0 restraints	Δρ_min_ = −0.26 e Å^−^^3^

### Special details

**Table:**

Experimental. The crystal was placed in the cold stream of an Oxford Cyrosystems Cobra open-flow nitrogen cryostat (Cosier & Glazer, 1986) operating at 100.0 (1) K.
Geometry. All e.s.d.'s (except the e.s.d. in the dihedral angle between two l.s. planes) are estimated using the full covariance matrix. The cell e.s.d.'s are taken into account individually in the estimation of e.s.d.'s in distances, angles and torsion angles; correlations between e.s.d.'s in cell parameters are only used when they are defined by crystal symmetry. An approximate (isotropic) treatment of cell e.s.d.'s is used for estimating e.s.d.'s involving l.s. planes.
Refinement. Refinement of *F*^2^ against ALL reflections. The weighted *R*-factor *wR* and goodness of fit *S* are based on *F*^2^, conventional *R*-factors *R* are based on *F*, with *F* set to zero for negative *F*^2^. The threshold expression of *F*^2^ > σ(*F*^2^) is used only for calculating *R*-factors(gt) *etc*. and is not relevant to the choice of reflections for refinement. *R*-factors based on *F*^2^ are statistically about twice as large as those based on *F*, and *R*- factors based on ALL data will be even larger.

### Fractional atomic coordinates and isotropic or equivalent isotropic displacement parameters (Å^2^)

**Table:**

	*x*	*y*	*z*	*U*_iso_*/*U*_eq_	
O1	0.22376 (12)	0.93012 (15)	0.99648 (5)	0.0209 (3)	
O2	0.11576 (12)	0.74263 (15)	1.04170 (5)	0.0193 (3)	
O3	0.51279 (12)	0.43230 (17)	0.85637 (5)	0.0263 (3)	
O4	0.43953 (12)	0.71714 (16)	0.86114 (5)	0.0226 (3)	
N1	0.28643 (14)	0.30918 (19)	0.97456 (6)	0.0185 (3)	
H1	0.2800	0.2043	0.9858	0.022*	
C1	0.12624 (18)	0.8380 (2)	0.84689 (7)	0.0239 (4)	
H1A	0.1920	0.9293	0.8559	0.029*	
C2	0.01109 (18)	0.8600 (3)	0.80172 (7)	0.0257 (4)	
H2A	0.0013	0.9654	0.7810	0.031*	
C3	−0.08953 (17)	0.7263 (3)	0.78718 (7)	0.0229 (4)	
C4	−0.07301 (18)	0.5727 (2)	0.81972 (7)	0.0245 (4)	
H4A	−0.1401	0.4829	0.8115	0.029*	
C5	0.04298 (17)	0.5511 (2)	0.86473 (7)	0.0216 (4)	
H5A	0.0523	0.4465	0.8858	0.026*	
C6	0.14465 (16)	0.6828 (2)	0.87865 (6)	0.0161 (3)	
C7	0.27351 (16)	0.6541 (2)	0.92694 (6)	0.0155 (3)	
H7A	0.3279	0.7655	0.9321	0.019*	
C8	0.23295 (15)	0.6169 (2)	0.98026 (6)	0.0147 (3)	
C9	0.23531 (15)	0.4472 (2)	1.00053 (6)	0.0162 (3)	
C10	0.36055 (16)	0.3373 (2)	0.93593 (7)	0.0172 (3)	
C11	0.36349 (15)	0.5037 (2)	0.91449 (7)	0.0164 (3)	
C12	−0.21263 (19)	0.7489 (3)	0.73743 (8)	0.0305 (4)	
H12A	−0.2578	0.6349	0.7279	0.046*	
H12B	−0.1818	0.7931	0.7068	0.046*	
H12C	−0.2765	0.8332	0.7460	0.046*	
C13	0.19301 (15)	0.7769 (2)	1.00645 (6)	0.0159 (3)	
C14	0.08916 (17)	0.8938 (2)	1.07421 (7)	0.0209 (4)	
H14A	0.0075	0.8693	1.0868	0.025*	
H14B	0.0716	1.0012	1.0514	0.025*	
C15	0.2111 (2)	0.9245 (3)	1.12300 (8)	0.0320 (5)	
H15A	0.1911	1.0216	1.1449	0.048*	
H15B	0.2907	0.9545	1.1104	0.048*	
H15C	0.2296	0.8170	1.1450	0.048*	
C16	0.44652 (16)	0.5398 (2)	0.87521 (7)	0.0190 (3)	
C17	0.50195 (18)	0.7686 (3)	0.81750 (7)	0.0264 (4)	
H17A	0.5882	0.7034	0.8220	0.032*	
H17B	0.5231	0.8962	0.8204	0.032*	
C18	0.4070 (2)	0.7290 (3)	0.76142 (8)	0.0313 (4)	
H18A	0.4428	0.7840	0.7335	0.047*	
H18B	0.3165	0.7766	0.7592	0.047*	
H18C	0.4008	0.6012	0.7557	0.047*	
C19	0.19215 (18)	0.3867 (2)	1.05056 (7)	0.0200 (3)	
H19A	0.2343	0.4630	1.0814	0.030*	
H19B	0.2214	0.2646	1.0592	0.030*	
H19C	0.0932	0.3939	1.0431	0.030*	
C20	0.43324 (17)	0.1719 (2)	0.92385 (7)	0.0226 (4)	
H20A	0.4138	0.1559	0.8846	0.034*	
H20B	0.4011	0.0688	0.9399	0.034*	
H20C	0.5313	0.1852	0.9394	0.034*	

### Atomic displacement parameters (Å^2^)

**Table:**

	*U*^11^	*U*^22^	*U*^33^	*U*^12^	*U*^13^	*U*^23^
O1	0.0275 (6)	0.0144 (6)	0.0225 (6)	0.0008 (5)	0.0094 (5)	0.0009 (5)
O2	0.0255 (6)	0.0153 (6)	0.0205 (6)	−0.0024 (5)	0.0120 (5)	−0.0047 (5)
O3	0.0255 (6)	0.0317 (8)	0.0250 (7)	0.0015 (5)	0.0126 (5)	−0.0034 (6)
O4	0.0257 (6)	0.0240 (7)	0.0217 (6)	−0.0031 (5)	0.0124 (5)	0.0043 (5)
N1	0.0245 (7)	0.0110 (7)	0.0214 (7)	−0.0001 (5)	0.0086 (6)	0.0007 (6)
C1	0.0240 (8)	0.0217 (9)	0.0259 (9)	−0.0020 (7)	0.0066 (7)	0.0049 (8)
C2	0.0272 (9)	0.0271 (10)	0.0233 (9)	0.0056 (7)	0.0076 (7)	0.0110 (8)
C3	0.0217 (8)	0.0322 (10)	0.0157 (8)	0.0069 (7)	0.0063 (6)	−0.0008 (7)
C4	0.0245 (8)	0.0242 (10)	0.0229 (9)	−0.0027 (7)	0.0031 (7)	−0.0037 (8)
C5	0.0267 (8)	0.0185 (9)	0.0184 (8)	−0.0019 (7)	0.0038 (7)	0.0026 (7)
C6	0.0184 (7)	0.0171 (8)	0.0146 (7)	0.0022 (6)	0.0076 (6)	−0.0006 (6)
C7	0.0180 (7)	0.0132 (8)	0.0159 (7)	−0.0017 (6)	0.0057 (6)	0.0001 (6)
C8	0.0164 (7)	0.0147 (8)	0.0127 (7)	−0.0010 (6)	0.0034 (6)	−0.0010 (6)
C9	0.0167 (7)	0.0165 (8)	0.0147 (7)	−0.0021 (6)	0.0033 (6)	−0.0011 (6)
C10	0.0170 (7)	0.0171 (8)	0.0172 (8)	−0.0016 (6)	0.0042 (6)	−0.0040 (7)
C11	0.0164 (7)	0.0168 (8)	0.0163 (8)	−0.0005 (6)	0.0047 (6)	−0.0031 (6)
C12	0.0288 (9)	0.0388 (12)	0.0212 (9)	0.0094 (8)	0.0023 (7)	0.0010 (8)
C13	0.0148 (7)	0.0181 (8)	0.0134 (7)	−0.0008 (6)	0.0015 (6)	0.0011 (6)
C14	0.0245 (8)	0.0176 (9)	0.0239 (9)	0.0005 (7)	0.0122 (7)	−0.0041 (7)
C15	0.0378 (11)	0.0348 (12)	0.0213 (9)	0.0069 (9)	0.0042 (8)	−0.0089 (8)
C16	0.0156 (7)	0.0243 (9)	0.0158 (8)	−0.0028 (6)	0.0022 (6)	−0.0029 (7)
C17	0.0266 (9)	0.0334 (11)	0.0219 (9)	−0.0067 (8)	0.0114 (7)	0.0030 (8)
C18	0.0316 (10)	0.0387 (12)	0.0233 (10)	−0.0062 (8)	0.0071 (8)	0.0032 (8)
C19	0.0271 (8)	0.0146 (8)	0.0195 (8)	−0.0006 (7)	0.0086 (7)	0.0015 (7)
C20	0.0218 (8)	0.0193 (9)	0.0275 (9)	0.0026 (7)	0.0079 (7)	−0.0015 (7)

### Geometric parameters (Å, °)

**Table:**

O1—C13	1.223 (2)	C9—C19	1.502 (2)
O2—C13	1.3463 (19)	C10—C11	1.351 (2)
O2—C14	1.454 (2)	C10—C20	1.500 (2)
O3—C16	1.213 (2)	C11—C16	1.475 (2)
O4—C16	1.361 (2)	C12—H12A	0.9600
O4—C17	1.450 (2)	C12—H12B	0.9600
N1—C9	1.385 (2)	C12—H12C	0.9600
N1—C10	1.386 (2)	C14—C15	1.498 (2)
N1—H1	0.8370	C14—H14A	0.9700
C1—C6	1.386 (2)	C14—H14B	0.9700
C1—C2	1.394 (2)	C15—H15A	0.9600
C1—H1A	0.9300	C15—H15B	0.9600
C2—C3	1.392 (3)	C15—H15C	0.9600
C2—H2A	0.9300	C17—C18	1.502 (2)
C3—C4	1.387 (3)	C17—H17A	0.9700
C3—C12	1.511 (2)	C17—H17B	0.9700
C4—C5	1.396 (2)	C18—H18A	0.9600
C4—H4A	0.9300	C18—H18B	0.9600
C5—C6	1.387 (2)	C18—H18C	0.9600
C5—H5A	0.9300	C19—H19A	0.9600
C6—C7	1.531 (2)	C19—H19B	0.9600
C7—C11	1.520 (2)	C19—H19C	0.9600
C7—C8	1.524 (2)	C20—H20A	0.9600
C7—H7A	0.9800	C20—H20B	0.9600
C8—C9	1.357 (2)	C20—H20C	0.9600
C8—C13	1.465 (2)		
			
C13—O2—C14	116.54 (13)	H12A—C12—H12C	109.5
C16—O4—C17	116.64 (14)	H12B—C12—H12C	109.5
C9—N1—C10	123.54 (14)	O1—C13—O2	122.08 (15)
C9—N1—H1	117.3	O1—C13—C8	123.33 (15)
C10—N1—H1	118.7	O2—C13—C8	114.57 (14)
C6—C1—C2	121.28 (17)	O2—C14—C15	110.15 (14)
C6—C1—H1A	119.4	O2—C14—H14A	109.6
C2—C1—H1A	119.4	C15—C14—H14A	109.6
C3—C2—C1	120.86 (16)	O2—C14—H14B	109.6
C3—C2—H2A	119.6	C15—C14—H14B	109.6
C1—C2—H2A	119.6	H14A—C14—H14B	108.1
C4—C3—C2	117.94 (16)	C14—C15—H15A	109.5
C4—C3—C12	121.34 (17)	C14—C15—H15B	109.5
C2—C3—C12	120.72 (17)	H15A—C15—H15B	109.5
C3—C4—C5	120.87 (16)	C14—C15—H15C	109.5
C3—C4—H4A	119.6	H15A—C15—H15C	109.5
C5—C4—H4A	119.6	H15B—C15—H15C	109.5
C6—C5—C4	121.24 (16)	O3—C16—O4	122.14 (15)
C6—C5—H5A	119.4	O3—C16—C11	127.33 (16)
C4—C5—H5A	119.4	O4—C16—C11	110.53 (14)
C1—C6—C5	117.77 (15)	O4—C17—C18	111.28 (14)
C1—C6—C7	121.68 (15)	O4—C17—H17A	109.4
C5—C6—C7	120.54 (14)	C18—C17—H17A	109.4
C11—C7—C8	111.04 (13)	O4—C17—H17B	109.4
C11—C7—C6	111.12 (13)	C18—C17—H17B	109.4
C8—C7—C6	110.72 (12)	H17A—C17—H17B	108.0
C11—C7—H7A	107.9	C17—C18—H18A	109.5
C8—C7—H7A	107.9	C17—C18—H18B	109.5
C6—C7—H7A	107.9	H18A—C18—H18B	109.5
C9—C8—C13	124.37 (14)	C17—C18—H18C	109.5
C9—C8—C7	121.06 (14)	H18A—C18—H18C	109.5
C13—C8—C7	114.56 (14)	H18B—C18—H18C	109.5
C8—C9—N1	118.86 (15)	C9—C19—H19A	109.5
C8—C9—C19	127.69 (15)	C9—C19—H19B	109.5
N1—C9—C19	113.42 (14)	H19A—C19—H19B	109.5
C11—C10—N1	119.30 (14)	C9—C19—H19C	109.5
C11—C10—C20	127.24 (15)	H19A—C19—H19C	109.5
N1—C10—C20	113.44 (14)	H19B—C19—H19C	109.5
C10—C11—C16	120.59 (15)	C10—C20—H20A	109.5
C10—C11—C7	120.89 (14)	C10—C20—H20B	109.5
C16—C11—C7	118.37 (14)	H20A—C20—H20B	109.5
C3—C12—H12A	109.5	C10—C20—H20C	109.5
C3—C12—H12B	109.5	H20A—C20—H20C	109.5
H12A—C12—H12B	109.5	H20B—C20—H20C	109.5
C3—C12—H12C	109.5		
			
C6—C1—C2—C3	0.2 (3)	C9—N1—C10—C11	−12.4 (2)
C1—C2—C3—C4	1.4 (3)	C9—N1—C10—C20	166.21 (14)
C1—C2—C3—C12	−178.65 (17)	N1—C10—C11—C16	177.23 (14)
C2—C3—C4—C5	−1.7 (3)	C20—C10—C11—C16	−1.1 (2)
C12—C3—C4—C5	178.29 (16)	N1—C10—C11—C7	−7.3 (2)
C3—C4—C5—C6	0.6 (3)	C20—C10—C11—C7	174.40 (15)
C2—C1—C6—C5	−1.4 (3)	C8—C7—C11—C10	22.6 (2)
C2—C1—C6—C7	177.57 (15)	C6—C7—C11—C10	−101.07 (17)
C4—C5—C6—C1	1.0 (2)	C8—C7—C11—C16	−161.75 (13)
C4—C5—C6—C7	−177.94 (15)	C6—C7—C11—C16	74.54 (17)
C1—C6—C7—C11	−112.17 (17)	C14—O2—C13—O1	−9.6 (2)
C5—C6—C7—C11	66.73 (19)	C14—O2—C13—C8	172.05 (13)
C1—C6—C7—C8	123.94 (17)	C9—C8—C13—O1	160.80 (15)
C5—C6—C7—C8	−57.15 (19)	C7—C8—C13—O1	−19.1 (2)
C11—C7—C8—C9	−21.7 (2)	C9—C8—C13—O2	−20.9 (2)
C6—C7—C8—C9	102.23 (17)	C7—C8—C13—O2	159.24 (13)
C11—C7—C8—C13	158.16 (13)	C13—O2—C14—C15	−80.96 (18)
C6—C7—C8—C13	−77.91 (16)	C17—O4—C16—O3	7.0 (2)
C13—C8—C9—N1	−174.47 (14)	C17—O4—C16—C11	−172.52 (13)
C7—C8—C9—N1	5.4 (2)	C10—C11—C16—O3	3.5 (3)
C13—C8—C9—C19	3.4 (3)	C7—C11—C16—O3	−172.17 (15)
C7—C8—C9—C19	−176.74 (15)	C10—C11—C16—O4	−177.07 (14)
C10—N1—C9—C8	13.3 (2)	C7—C11—C16—O4	7.31 (19)
C10—N1—C9—C19	−164.90 (14)	C16—O4—C17—C18	80.78 (19)

### Hydrogen-bond geometry (Å, °)

**Table:**

*D*—H···*A*	*D*—H	H···*A*	*D*···*A*	*D*—H···*A*
N1—H1···O1^i^	0.84	2.15	2.9684 (18)	166

Symmetry codes: (i) *x*, *y*−1, *z*.
